# Dance Is an Accessible Physical Activity for People with Parkinson's Disease

**DOI:** 10.1155/2021/7516504

**Published:** 2021-10-22

**Authors:** Sara Emmanouilidis, Madeleine E. Hackney, Susan C Slade, Hazel Heng, Dana Jazayeri, Meg E. Morris

**Affiliations:** ^1^Academic and Research Collaborative in Health, La Trobe University, Bundoora, Victoria, Australia; ^2^Emory University School of Medicine, Department of Medicine, Division of Geriatrics and Gerontology, Atlanta, USA; ^3^Atlanta Veterans Affairs Centre for Visual & Neurocognitive Rehabilitation, Decatur, GA, USA; ^4^Victorian Rehabilitation Centre, Glen Waverley, Victoria, Australia

## Abstract

**Objective:**

To evaluate the outcomes of face-to-face, digital, and virtual modes of dancing for people living with Parkinson's disease (PD).

**Design:**

Systematic review informed by Cochrane and PRIMSA guidelines. *Data Sources.* Seven electronic databases were searched: AMED, Cochrane, PEDro, CINHAL, PsycINFO, EMBASE, and MEDLINE.

**Methods:**

Eligible studies were randomised controlled trials (RCT) and other trials with quantitative data. The PEDro scale evaluated risk of bias for RCTs. Joanna Briggs Institute instruments were used to critically appraise non-RCTs. The primary outcome was the feasibility of dance interventions, and the secondary outcomes included gait, balance, quality of life, and disability.

**Results:**

The search yielded 8,327 articles after duplicates were removed and 38 met the inclusion criteria. Seven were at high risk of bias, 20 had moderate risk of bias, and 11 had low risk of bias. There was moderately strong evidence that dance therapy was beneficial for balance, gait, quality of life, and disability. There was good adherence to digital delivery of dance interventions and, for people with PD, online dance was easy to access.

**Conclusion:**

Dancing is an accessible form of exercise that can benefit mobility and quality of life in people with PD. The COVID-19 pandemic and this review have drawn attention to the benefits of access to digital modes of physical activity for people living with chronic neurological conditions.

## 1. Introduction

Parkinson's disease (PD) is a debilitating and progressive condition that currently has no cure. People living with PD can experience movement disorders and nonmotor symptoms that compromise their levels of physical activity [1] and quality of life [2–5]. Movement slowness [6], balance impairment [7], falls [8, 9], and gait disturbance often occur [10]. These movement disorders, coupled with anxiety, depression, or lethargy, can be major barriers to maintaining long-term engagement in physical activity [1].

Structured exercises and physical activities can assist people with chronic diseases to keep moving and to stay engaged in social activities and recreational sports [1]. One of the challenges for practitioners is keeping people with Parkinson's motivated to adhere to regular physical activities over long periods of time [1]. People typically live with PD for 7–25 years [11, 12], and clinical guidelines recommend daily physical activities for at least 30–45 minutes per session [13]. For progressive conditions such as PD, it is recommended that a range of therapeutic exercises is available, to maintain long-term exercise adherence and compliance [1, 14]. There is evidence that people in the early to midstages of Parkinson's can benefit from progressive resistance strength training [8, 15, 16], cueing [10,15], aqua therapy [17], physiotherapy [18, 19], aerobic exercises [20], Nordic walking [21], community walking [22], assisted cycling [23], boxing [24], and tai chi [25]. Therapeutic dancing is another option, given that it is engaging and can be done in groups or individually [26–28].

Dancing for people with PD aims to improve movement, wellbeing, and quality of life, as well as social engagement and exercise capacity [29–32]. Dance also allows for creative expression and can take the focus off the disease and onto movement to music and social connection [31,33,34]. A study by Dos Santos Delabary et al. [35] reported that dance sometimes has greater benefits for functional mobility and motor symptoms than usual care. Likewise, Shanahan et al. [36] noted that participation in dance can improve endurance, motor impairment, and balance for those with mild to moderate PD. Berti et al. [37] reported that adapted tango dance programs are an effective intervention for individuals with PD with a range of abilities and balance limitations.

Given the need for people with Parkinsonism and related disorders to have a range of evidence-based exercise choices, the primary aim was to evaluate the outcomes of face-to-face, online, and virtual modes of therapeutic dancing as an accessible physical activity for people living with PD. The outcomes of particular interest were balance, gait, disability, and quality of life.

## 2. Methods

We conducted a systematic review of the literature following *a priori* methods. Two independent reviewers (SE, HH) were involved in the selection of studies into the review and two independent reviewers (SE, DJ) completed the data extraction to ensure that all relevant studies were identified, and that data were extracted reliably and consistently [38]. The review was informed by Cochrane guidelines and reported according to the Preferred Reporting Items for Systematic Reviews and Meta-Analysis (PRISMA) checklist [39].

The search was conducted using seven electronic databases: AMED, Cochrane, PEDro, CINHAL, PsycINFO, EMBASE, and MEDLINE. The search terms included: Parkinson disease or Parkinson's disease or Parkinson∗, movement disorders and dance therapy or dancing or dance based or danc∗ or foxtrot or tango or waltz or “Irish set” or ballroom or dance movement therapy or contemporary salsa or cultural and telerehabilitation or telemedicine or telehealth or tele or remote or online or web-based or virtual or in-person or pre-recorded or live or synchronous or asynchronous or partnered or on-partnered and quality of life or balance or gait or disability. The MEDLINE strategy was adapted to the other databases and search strategies are available on request. An example of the Medline search strategy is in Table 1. The searches were conducted by a health sciences librarian up until June 2020, saved in each database, and downloaded into the bibliographic management software program Endnote [40, 41]. Search yields were combined into one Endnote library, and duplicates were deleted prior to application of the eligibility criteria to the titles.

Inclusion and exclusion criteria were firstly applied to the titles to exclude studies that were clearly ineligible. We then applied the eligibility criteria to the titles and abstracts (SE, HH). Two reviewers (SE, HH) independently read in full the remaining articles to determine whether they met the eligibility criteria. A third reviewer (SS) was consulted to reach consensus if needed. Remaining discrepancies were resolved through consensus by two final reviewers (MM, MH) to determine the final included studies.

### 2.1. Inclusion Criteria

#### 2.1.1. Study Designs

The study designs included randomised controlled trials (RCT) and nonrandomised trials that contained data. We deliberately included both randomised and nonrandomised trials, to extend the findings of systematic reviews, which were confined to RCTs (e.g., 35–37). The full text had to be available and accessible in English. Systematic reviews, meta-analyses, protocol papers, letters to the editor, conference posters, opinion pieces, and abstracts were excluded.

#### 2.1.2. Participants

Participants had to have a diagnosis of PD. Other chronic neurological, musculoskeletal, or respiratory conditions were excluded, as well as dementia and Alzheimer's disease. Individuals were at any stage of PD classified by the modified Hoehn and Yahr Scale [42] and living in residential care or the community. Adults of all ages, genders, and many cultures were included.

#### 2.1.3. Interventions

Studies were included if they used dance as an exercise intervention or form for physical activity or physiotherapy. All genres of dance were eligible, including Irish-set dancing, tango, waltz, tap, jazz, salsa, ballroom, ballet, mixed genre, and creative dancing. Classes could be delivered partnered or nonpartnered, group or one to one and with or without music. The mode of delivery was in-person, digitally (also known as online), or using virtual tools. Online delivery was via platforms such as Zoom® or Microsoft Teams®. Some of the dance interventions were delivered by dance teachers and others were delivered using “virtual” tools such as Wii (Nintendo Inc., Japan) or Sony Play Station® video game systems. The criteria for comparison or control interventions were any “usual care” or “usual physical activity” condition or any other therapeutic intervention.

#### 2.1.4. Outcomes

Studies were selected if they included baseline and after intervention outcomes for any of the following variables: gait, balance, movement, mobility, movement disorders, nonmotor symptoms, disability, participation, quality of life, wellbeing, or social participation. Feasibility studies were also reviewed.

#### 2.1.5. Risk of Bias

The PEDro scale was used to determine the risk of bias for RCTs [43]. PEDro was selected as it is a valid and reliable appraisal instrument for RCTs [44, 45]. Joanna Briggs Institute (JBI) instruments were used to critically appraise nonrandomised studies and to determine their risk of bias [46, 47]. Risk of bias assessments were completed independently by two reviewers (SE, SS), and consensus was reached by consultation with the research team (MM, MH).

#### 2.1.6. Data Extraction

Reviewers (SE, DJ,) independently extracted data into a pretested spreadsheet under headings such as study, participant and intervention characteristics, and outcome data. The data were independently screened and confirmed (SS, MEM). Outcome data were extracted for short-, medium-, and long-term follow-up assessments when reported.

#### 2.1.7. Data Analysis

For quantitative data, summary statistics were calculated. For the RCTs, the reported means and standard deviations were tabulated, and the Hedge's g, bias-corrected effect size (ES) index was used to estimate the effects of dancing compared to another intervention or no therapy [48]. In some cases, the ES was already reported as a standardized mean difference (SMD) or Cohen's d [49, 50] which we used. The Hedge's g and Cohen's d are similar; the Hedge's g tends to perform better with sample sizes lower than 20. Whenever possible, a 95% confidence interval (95% CI) was calculated around the SMD for an estimate of the range of intervention effects [51]. Median scores and interquartile ranges (IQR), reported by the study authors, were also tabulated [52]. To facilitate comparisons across studies, median scores were entered into SMD calculations as best estimates of mean scores [53]. For non-RCTs, within-group mean differences and change scores were reported and effect sizes calculated whenever possible.

## 3. Results

**Figure 1:**
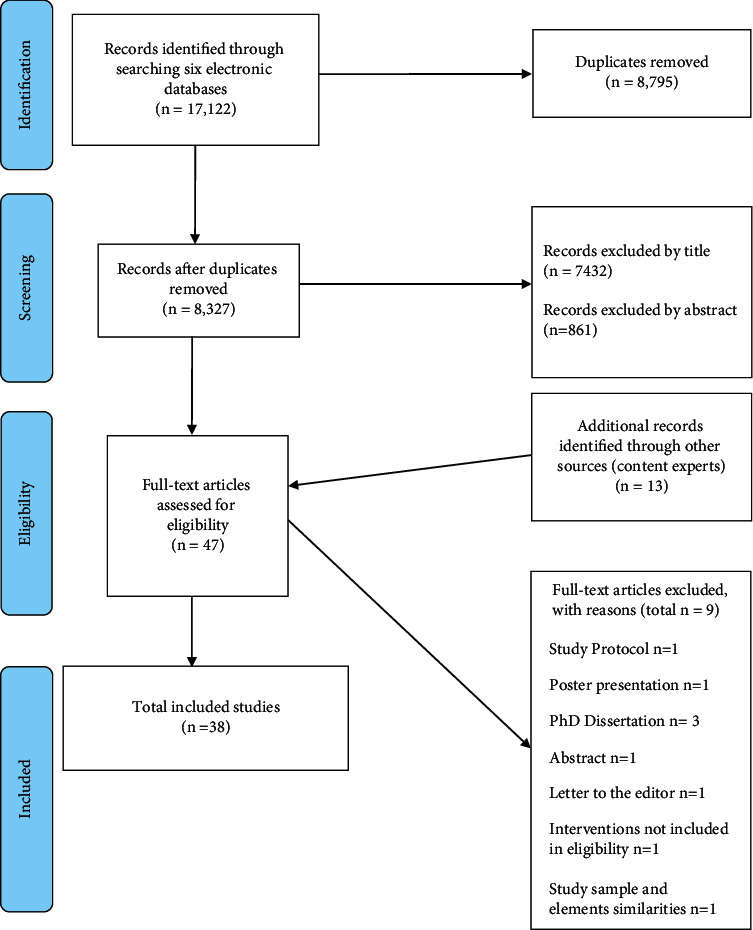
PRISMA flow diagram.

Of the initial yield of 17,122, there were 8,327 remaining after duplicates were removed. Screening of the articles was conducted by two independent reviewers (SE, HH) with 34 articles initially assessed for eligibility. A third reviewer (SS) was consulted to check the findings and reach consensus and 13 additional articles were added by members of the research team (MM, MH). From the articles read in full, 9 were excluded because they did not meet the eligibility criteria. Final consensus was reached in consultation with MM and MH, yielding a total of 38 articles.  Figure 1 shows the PRISMA-compliant flowchart for selection of studies [39]. Of the included studies, 17 were RCTs [26, 27, 30, 54–67]. One of these was a sequential RCT [66], one was a quasi-RCT [67], and one was an RCT with a crossover design [63] (Table 2). Of the trials, 21 had nonrandomised designs [28, 68–87], and one of these was a quasiexperimental study [81]. One used mixed methods design [82] for which quantitative data were extracted and analysed. Also, one was an exploratory trial [87] and there was an additional single case study [88]. (Table 2).

The included studies ranged in sample size from 6 to 96 participants (Table 2). Only 3 studies included either telehealth [84] or technology-based interventions that included virtual reality dancing [57] or dance Google glass modules [73]. Dance interventions included tango [27, 28, 54, 55, 58, 59, 60–62, 64, 66, 68, 70, 72, 80, 82, 86], Sardinian folk dancing [56], Irish set dancing [26, 65], waltz/foxtrot [55, 61], ballet [85], Brazilian Samba [83], Zumba [71], Qigong dance [63], improvisation dance [79], or mixed dance genres [27, 55, 62, 66, 67, 77, 78], with three studies including home-based dance programs [27, 68, 76]. The duration of interventions ranged from two weeks to two years with frequency per week varying from once a week to daily. The intervention session length was usually 1 hour, although it ranged from 30 minutes to two hours.

Method quality and risk of bias assessments were conducted for all studies.  Table 3 shows that the risk of bias for five of the RCTs was high [54, 57, 59, 61, 66]. It was also high for two of the nonrandomised studies [72, 75]. In addition, 20 were at moderate risk of bias (eight RCTS, 12 nonrandomised studies) [26, 28, 30, 55, 56, 58, 65, 67, 69–71, 73, 74, 76–79, 84, 87, 88] and 11 were at low risk of bias (four RCTs, seven nonrandomised studies) [27, 62–64, 68, 81–83, 85, 86, 80]. For RCTs, blinding of the participants and therapists was generally not possible due to the nature of dance therapy. A large number of RCTs did not include intention to treat analysis [26, 30, 54–57, 59, 61, 65–67], concealed allocation [30, 54–61, 66], or reporting of outcomes for more than 85% of participants at each time point [26, 54, 57, 59–62, 65–67]. These omissions increased the risk of bias (Table 3). Non-RCT studies were identified as having increased risk of bias as there was no control group [28, 68, 70–74, 75–80, 84, 87], or they did not receive similar treatment or care [28, 68–75, 80, 81, 84, 87], or they did not conduct a follow-up [69–72, 74–76, 83–85, 87].

Data analysis is presented in Tables 4 and 5. Overall, the results showed moderate to large benefits from therapeutic dance for people with mild to moderate PD (Table 4). RCTs demonstrated significant short-term benefits for balance with the Berg Balance scale (BBS) [30, 56, 57, 59–61, 67], significant reduction in disability measured by the Unified Parkinson's Disease Rating Scale (UPDRS) [26, 30, 56, 59, 61, 62, 65, 67], significantly improved mobility measured by Timed Up and Go (TUG) [56, 58, 59, 61], significantly improved endurance measured by the 6 Minute Walk Test [56, 61], significantly reduced gait freezing measured by the Freezing of Gait scale [59, 61, 65], and significantly reduced depression measured by the Beck Depression Inventory [57]. Meta-analysis was not conducted due to intervention and outcome measure heterogeneity.

The effects of dancing for PD reported in non-RCTs demonstrated end of intervention benefits for people with mild to moderately severe disease (Table 5). There were improvements in balance (BBS) [28, 72, 77, 80], disability (UPDRS) [68, 72, 78, 80, 86], mobility (TUG) [28, 72, 73, 75, 80] and Tinetti Mobility Scale [75], depression [70, 83], and quality of life [26, 68–70, 86, 87]. Key studies on digital delivery modes for dancing with PD (e.g., [57, 73, 84]) showed that virtual technologies can be an accessible and beneficial method of physical activity for some people living with this chronic and progressive disease.

## 4. Discussion

This systematic review of the global literature showed that dancing for individuals with mild to moderately severe PD could be a beneficial and accessible form of physical activity for some people, whether delivered face-to-face or using an online telemedicine platform or “virtual dance” video-gaming tools. The findings support mounting evidence that therapeutic dance can, in the short term, significantly improve balance, mobility, gait, disability, and quality of life in PD [26, 68, 70, 86, 87, 89]. Although the recruitment levels in the reviewed studies did not always meet clinical trial targets, attendance and adherence to dance classes were generally high. The duration of the dancing classes and session lengths varied, and improvements were seen in interventions running for two weeks [72] up to 2 years [54]. There were significant improvements when session lengths ranged from 30 to 90 minutes per day. Although previous systematic reviews of dance for Parkinson's disease were conducted by Shanahan et al. (2017) [36], Carapellotti et al. [31], Berti et al. [37], and Rocha et al. [90], all of those were confined to randomised controlled clinical trials. By conducting a more recent search and extending our analysis to RCTs and non-RCT quantitative studies, our review captured more of the therapies currently being implemented in clinical practice.

Our review suggests good attendance for the telehealth mode of delivery in chronic diseases, possibly because digital delivery reduces geographical, environmental, economic, and commute barriers [91]. Some technological difficulties can be encountered with digital delivery [84] such as Internet and usability problems and the need for training and guidance in how to operate the technology. Nevertheless, the reviewed articles did not directly analyse the risk of falls with online delivery or when using video modes. For people with moderate to advanced disease, postural instability and falls can be problematic. Precautions need to be taken to ensure that people at home have strategies to prevent and manage falls, should they occur.

There are several clinical implications of this systematic review. Dancing was shown to be clinically feasible, with high levels of adherence by participants and considerable interest in future classes. Many of the publications that we reviewed supported the need to increase access to community dance classes to improve exercise capacity and wellbeing, as well as to increase the opportunity for people living with Parkinsonism to socialise. For face-to-face dance classes, there is a need to determine how to reduce barriers to participation, including transportation, access, and cost, (please see [1, 92]). For digital modes of dancing, clinical protocols are needed to support safe and sustainable implementation and guidance in the use of technology. In addition, it could be argued that a need exists for credentialing programs for dance teachers and practitioners, to ensure evidence-based and effective delivery of this form of structured exercise [93] as well as protocols for clinicians and dance teachers to ensure safe and effective delivery. In the current global context of the COVID-19 pandemic, there is arguably a need to further explore digitally delivered dance and other forms of physical activity for people living with chronic neurological conditions [91, 94].

Despite the systematic review being informed by the Cochrane guidelines, there were several limitations. Most studies included people with mild-moderate disease. The findings might not generalise to people with end-stage disease or very old people, or those who cannot access face-to face classes or digital technologies. Many trials did not control for the effects of levodopa or other Parkinson's medications. Intervention duration and frequency were reported; however, none of the studies documented the intensity of dance therapy. Although some large, significant effects were demonstrated, these need to be considered in light of the moderate to high risk of bias in many studies, especially those that were not randomised trials. Although dance and music were shown to have benefit for people living with PD, the most effective dance genre or music type require further exploration [95]. Quality of life can be adversely affected by Parkinsonism [3, 5], and the mechanisms by which arts-health therapies such as dance and music can improve health-related quality of life warrant further exploration [95]. Also, the reviewed articles were in the English-language, limiting generalisability to non-English speaking cultures and their associated dance genres.

To conclude, dance is safe and feasible for some people in the early to midstages of PD, provided that safety precautions and training are incorporated into design and delivery. There are positive associations between therapeutic dancing and improvements in gait, balance, movement disorders, and disability. For some individuals, there can be improvements in quality of life. There was preliminary evidence that delivery of dancing for PD online is beneficial for some people, although there is a need to verify the efficacy and safety of this modality, especially for people who are frequent fallers.

## Figures and Tables

**Table 1:** Medline search.

Search ID#	Search terms	Search notes	Results
1	Exp Parkinson disease, secondary/or exp Parkinson disease/		70995
2	Parkinson∗.mp.		127920
3	Exp stroke/or exp stroke rehabilitation/		136433
4	(Stroke or strokes).mp.		295325
5	Exp Multiple sclerosis/		58198
6	“Multiple sclerosis”.mp.		82515
7	1 or 2 or 3 or 4 or 5 or 6		519376
8	Exp dance therapy/		359
9	Exp dancing/		2848
10	(Dance∗ or dance-based or dancing).mp.		7345
11	(Foxtrot or tango or Waltz or “Irish set” or ballroom or ballet).mp.		2113
12	Or/8–11	Dance related terms	8348
13	Exp exercise movement techniques/		8116
14	physiotherap∗.mp.		26124
15	Exp exercise therapy/		50130
16	Exp exercise/		192926
17	exercis∗.ti,ab.		290086
18	Or/13–17	Exercise/physio related terms	431332
19	12 or 18	Included dance and physio/exercise terms	438077
20	Exp telemedicine/or exp telerehabilitation/		27950
21	telerehabilitation.mp.		933
22	tele.mp.		3173
23	(Remote adj3 rehabilitat∗).mp.		87
24	telehea∗.mp.		4922
25	app.mp.		25736
26	((Exercise or mode) adj2 delivery).mp.		8376
27	virtual.mp.		59008
28	video.mp. or exp video recording/or tape recording/or videotape recording/		148947
29	(Online or “online”).ti,ab.		137403
30	telemedicine.mp.		27556
31	Exp telemedicine/		27950
32	(Telemonitor∗ or tele-monitor∗).mp.		1706
33	Internet.mp.		102501
34	((Tele∗ adj2 coach∗) or telecoach∗).mp.		374
35	videoconferenc∗.mp.		2843
36	ipad.mp.		1300
37	computer.mp.		693528
38	Exp internet/or exp internet-based intervention/		78406
39	Mobile applications/		5683
40	(Apps or “mobile applications”).mp.		10631
41	zoom.mp.		1600
42	webinar∗.mp.		569
43	(Live adj2 stream∗).mp.		148
44	Web-based.mp.		30097
45	Pre-record∗.mp.		363
46	(Dvd adj2 deliv∗).mp.		31
47	Or/20–46		1110851
48	7 and 19 and 47	Final results	1903
49	recorded.mp.		525831
50	synchronous.mp.		35231
51	asynchronous.mp.		9988
52	49 or 50 or 51		566263
53	7 and 19 and 52	Testing results for synchronous/asynchronous.	
Or recorded	1204		

**Table 2:** Study characteristics.

First author, year	Study design		Sample size	Interventions	Age (years) mean ± SD Sex (M, F)	H&Y mean ± SD, range, or median (IQR)	Session length, frequency, intervention duration	Medications	Outcome measures
*Randomised studies*									
Duncan and Earhart, 2012 [62]	RCT		62	Argentine Tango	69.3 ± 1.9 M: 15, F: 11	2.6 ± 0.1	1 hr class, 2/week, 12 months	Not reported but tested in the “off phase”	UPDRS-II, UPDRS-III, FOG, 6MWT, MiniBEST, gait velocity
				Control, no dance	69.0 ± 1.5 M: 15, F: 11	2.5 ± 0.1			
Duncan and Earhart, 2014 [54]	RCT		10	Argentine Tango	67.8 ± 8.72 M: 4, F: 1	2.4	1 hr, 2/week, 2 years	Levodopa	UPDRS-I, UPDRS-II, UPDRS-III, MiniBEST, gait velocity, TUG, dual-task TUG, 6MWT, FOGQ
				Control, no dance	66 ± 11.0 M: 4, F: 1	2.3			
Kunkel et al., 2017 [55]	RCT		51	Dance (mixed genre)	71.3 ± 7.7 M: 19, F: 17	1–3	1 hr, 2/week, 10 weeks	Not reported	BBS, SS180, TUG, 6MWT, Phone-FITT, EQ-5D, ABC
				Control, no dance	69.7 ± 6.0 M: 6, F: 9	1–3			
Hackney and Earhart, 2010 [60]	RCT		39	Partner dancing	69.6 ± 8.5 M: 13, F: 6	2-3	1 hr, 2/week, 10 weeks	Levodopa	UPDRS-III, BBS, tandem stance, one leg stance, TUG, 6MWT, gait measures
				Nonpartner dancing	69.6 ± 9.5 M: 15, F: 5	2-2.6			
Hackney and Earhart, 2009 [61]	RCT		58	Waltz/foxtrot	66.8 ± 2.4 M: 11, F: 6	2.0 ± 0.2	1 hr, 2/week, 13 weeks	Levodopa	UPDRS-III, BBS, TUG, 6MWT, gait measures, FOGQ
				Tango	68.2 ± 1.4 M: 11, F: 3	2.1 ± 0.1			
				Control, no dance	66.5 ± 2.8 M: 12, F: 5	2.2 ± 0.2			
Hackney et al., 2007 [59]	RCT		19	Tango	72.6 ± 2.20 M: 6, F: 3	2.3 ± 0.7	1 hr, 2/week, 13 weeks	PD medications	UPDRS-III, BBS, gait velocity, TUG, FOGQ
				Group exercise class	69.6 ± 2.1 M: 6, F: 4	2.2 ± 0.6			
Hashimoto et al., 2015 [67]	Quasi-RCT		46	PD dance	67.9 ± 7.0 M: 3, F: 12	Score 2 (11)	60 min class, 1/week, 12 weeks	Not reported	TUG, BBS, UPDRS, SDS, FAB, Mental Rotation Task, Apathy Scale
				PD exercise	62.7 ± 14.9 M: 2, F: 15	Score 3 (33)	60 min class, 1/week, 12 weeks		
				Control, usual care	69.7 ± 4.0 M: 7, F: 7	Score 4 (2)			
Lee et al., 2015 [57]	RCT		20	Virtual reality dance	68.4 ± 2.9 M: 5, F: 5	Not reported	30 mins, 5/week, 6 weeks	Not reported	BBS, BDI, MBI
				Control	70.1 ± 3.3 M: 5, F: 5				
Lee et al., 2018 [63]	RCT with cross-over design		32	Qigong dance	65.8 ± 7.2 M: 10, F: 15	1–3	60 min, 2/week, 8 weeks	Not reported	UPDRS, PDQL, BBS, BDI
				Control, wait list	65.7 ± 6.4 M: 7, F: 9	1–3			
McKee and Hackney, 2013 [66]	Sequential RCT		33	Tango	68.4 ± 7.5 M: 12, F: 12	2.3 (2.0-2.6)	90 mins, 20 sessions, 12 weeks	PD medications	UPDRS-III, Four-Square Step Test, TUG, dual-task TUG, PDQ-39, FOGQ, SFHS-12
				Lecture series	74.4 ± 6.5 M: 8, F: 1	2.0 (2.0- 2.0)			
Michels et al., 2018 [30]	Pilot RCT		13	Dance therapy	69.2 ± 8.7 (total) M: 6, F: 7 (total)	2.11 ± 0.33 2.5 ± 1.00	60 mins, 1/week, 10 weeks	Stable PD medication regimen	UPDRS, MOCA, TUG, BBS, BDI, FSS, Visual Analog Fatigue Scale, PDQ-39
				Control, support group					
Poier et al., 2019 [64]	Pilot RCT		29	Argentine Tango	68.50 ± 8.07 M: 9, F: 5	Not reported	60 min class, 1/week, 10 weeks	Not reported	PDQ-39, BMLSS, ICPH
				Control, Tai Chi	68.87 ± 10.96 M: 3, F: 12				
Rocha et al., 2018 [27]	Pilot RCT		21	Argentine Tango	70.2 ± 5.5 M: 4, F: 6	1–4	In-person: 1 hour, 1/week Home: 40 mins, 1/week, 8 weeks	PD medication	TUG, BBS, functional gait assessment, FOGQ, UPDRS-II and -III, PDQ-39
				Mixed dance	72.9 ± 5.5 M: 4, F: 7				
Rio Romenets et al., 2015 [58]	RCT		33	Argentine Tango	63.2 ± 9.9 M: 12, F: 6	2 ± 0.5	1 hr, 2/week, 12 weeks	Not reported	UPDRS-III, UPDRS, MiniBEST, TUG, dual-task TUG, BDI, Apathy Scale, KFSS, PDQ-39, CGI-C, FOGQ, Falls Questionnaire, MOCA
				Control	64.3 ± 8.1 M: 7, F: 8	1.7 ± 0.6			
Shanahan et al., 2017 [26]	RCT		41	Set dancing	69 ± 10 M: 13, F: 7	1.25 ± 1	In-person: 1 hr, 1/week	Not reported	UPDRS-III, 6MWT, MiniBEST, PDQ-39
				Control, usual care and ADL	69 ± 8 M: 13, F: 8	2 ± 1	Home: 20 mins, 3/week, 10 weeks		
Solla et al., 2019 [56]	RCT		20	Sardinian folk dance	67.8 ± 5.9 M: 6, F: 4	2.1 ± 0.6	90 mins, 2/week, 12 weeks	PD medications	UPDRS-III, 6MWT, BBS, TUG, Parkinson's Disease Fatigue Scale, BDI, Starkstein Apathy Scale, MOCA
				Control, usual care	67.1 ± 6.3 M: 7, F: 3	2.3 ± 0.4			
Volpe et al., 2013 [65]	RCT: single blind, parallel group		24	Intervention: Irish set dancing	61.6 ± 4.5 M: 7, F: 5	2.2 ± 0.4	90 min classes, 1/week, 6 months	Levodopa (n=24) I: 725.0 mg ± 234 C: 645.0 ± 216 Also, pramipexole,ropinirole, rotigotine,rasagiline, entacapone	UPDRS-III, BBS, FOG, PDQ-39
				Control: physiotherapy (balance, cueing, gait training, strength)	65.0 ± 5.3 M: 6, F: 6	2.2 ± 0.4	90 mins, 1/week, 6 months, 1 hr weekly home video		
*Nonrandomised studies*									
Albani et al., 2019 [68]		Single group, pre-post design, feasibility	10	Tango-based home exercises and group sessions	63.1 ± 9.25 M: 6, F: 4	2-3	Home: 1 hr, 4/week, 5 weeks Group session: 2 hr 1/ week, 5 weeks	Not reported	Gait measures (fully cued visual four-choice reaction-time tasks, simple reaction-time task, an uncued task, two partially cued tasks), UPDRS
Batson, 2010 [77]		Single group, pre-post design	11	Modern dance	72.7 + 8.7 M: 5, F: 6	1-2.5	85 min class 3/week, 3 weeks	Sinemet n=7, Azilect n=1, Requip n=1, No med n=2	TUG, FAB
Blandy et al., 2015 [70]		Single group, pre-post design	6	Argentine Tango	64 ± 6.28 M: 3, F: 3	2 (2-2)	1 hr, 2/week, 4 weeks	“ON” phase of medication	EQ-5D, Visual Analogue Scale, BDI
Dahmen-Zimmer and Jansen, 2017 [69]		Pre-post design, pilot trial	37	Karate (Shotokan)	68.87 ± 7.24 M: 13, F: 3	1–3	1 hr, 1/week, 30 weeks	Not reported	Multidimensional Mood State Questionnaire, Hospital Anxiety and Depression Scale, CEDS Depression Scale, SFHS-12, Short Scale Of General Self-Efficacy
				Dance (mixed genre)	72.33 ± 6.69 M: 6, F: 3				
				Control (waitlist)	70.42 ± 10.07 M: 8, F: 4				
Delextrat et al., 2016 [71]		Single group, pre-post design, feasibility	11	Zumba	64.0 ± 8.1 M: 5, F: 6	<3	45 mins–1 hr, 1/week, 6 weeks	Not reported	Enjoyment, change in physical activity, exercise intensity (accelerometry), heart rate, rated perceived exertion
Hackney and Earhart, 2009 [72]		Single group, pre-post design	14	Argentine Tango	67.2 ± 9.6 M: 9, F: 5	2.4 (25%:2.0; 75%:2.5)	1.5 hrs, 5/week, 2 weeks	Not reported	UPDRS-III, BBS, TUG, 6MWT, gait measures
Hackney and McKee, 2014 [28]		Single group, pre-post, pilot trial	88	Argentine Tango (PD)	68.4 ± 7.5 M: 13, F: 12	1–3	1.5 hrs, 2/week for 12 weeks	PD medication	UPDRS-III, BBS, TUG, gait speed assessment, 30 second chair stand, tandem stance
				Older adults (non-PD)	82.3 ± 8.8 M: 49, F: 14				
Heiberger et al., 2011 [78]		Single group, pre-post design	11	Mixed genre dance	71.3 ± 8.4 M: 5, F: 6	Moderate to severe 2.5–4	1/week class, 8 months	L-Dopa or dopamine agonists	UPDRS-III, TUG, QOLS, Westheimer Questionnaire
Kalyani et al., 2019 [81]		Quasiexperimental design	33	Dance (mixed genre)	65.24 ± 11.88 M: 3, F: 14	1.65 ± 0.79	1 hr, 2/week, 12 weeks	Not reported	UPDRS-I, UPDRS-II, PDQ39
				Control	66.50 ± 7.70 M: 10, F: 6	1.56 ± 0.81			
Listewnik and Ossowski, 2018 [75]		Single group, pre-post design	10	Dance	69.9 ± 6.47 M: 5, F: 5	—	70 mins, 2/week for 12 weeks	Not reported	Tinetti POMA Test, TUG, 6MWT
McGill et al., 2018 [85]		Non-RCT	32	Ballet	69.83 ± 4.55 M: 9, F: 10	2.32 ± 0.48	1.25-1.5 hrs, 1/week for 1 year	Not reported	Step and stride variability, ABC
				Control	73.25 ± 8.09 M: 6, F: 7	2.15 ± 0.55			
McKay et al., 2016 [80]		Single group study	22	Adapted Tango	65.4 ± 12.8 M: 7, F: 15	1–4	90 mins, 15 lessons over 3 weeks	Not reported	UPDRS, dyskinesia, BBS, Dynamic Gait Index, FAB, musculoskeletal health, 6MWT, single and dual-task TUG, fast and preferred cadence, ABC, FOGQ
McNeely et al., 2015 [74]		Pre-post design	16	Dancing for PD (mixed genre) Tango	68.25 ± 10.90 M: 4, F: 4	2.25 ± 0.27	1 hr group class, 2/week, 12 weeks	Levodopa	UPDRS-III, MiniBEST, TUG, dual-task TUG, 6MWT, gait velocity
					67.66 ± 8.62 M: 4, F: 4	2.13 ± 0.58			
McRae et al., 2018 [87]		Exploratory study, surveys	61	Dancing for Parkinson's Disease (mixed genre)	67 ± 13 M: 21, F: 40	1–4	1-2/week, 12 months (6months–2 years), session length unknown	Not reported	36-Item Short Form Health Survey, Self-Efficacy Scale, functional mobility
Marchant et al., 2010 [79]		Single group, pre-post design	11	Improvisation dance	71.2 ± 6.1 M: 4, F: 7	2.4 ± 0.4	10 1.5-hour classes, 2 weeks	PD medication	UPDRS-III, BBS, TUG, gait measures, Five Times Sit-to-Stand Test, 6MWT, FOGQ, ABC, PDQ-39
Rawson et al., 2019 [86]		Nonrandomised controlled	96	Tango vs treadmill vs control (stretching)	67.2 ± 8.9 M: 56, F: 40	1–4	1 hr class, 2/week, 12 weeks	Levodopa	UPDRS-III, PDQ-39, 6MWT, MiniBEST, gait measures
Seidler et al., 2017 [84]		Non-RCT	26	Telerehab dance	68.1 ± 7.9 M: 4, F: 6	1–3	1 hr, 2/week, 12 weeks	Not reported	MiniBEST, UPDRS-III, gait velocity
				In-person dance	68.9 ± 9.4 M: 5, F: 5				
Shanahan et al., 2015 [76]		Single group, pre-post pilot trial	10	In-person and home-based dance programs	66.66 ± 5.87 M: 7, F: 3	1.5 ± 0.5	In-person: 1.5 hrs. 1/week Home: 20 mins, 2/week, 8 weeks	Not reported	UPDRS, 6MWT, BBS
Tillmann et al., 2020 [83]		Non-RCT	47	Brazilian samba	67 ± 9.2 n=23	1.8 ± 0.7	1 hr, 2/week, 12 weeks	4 months stable medication	PDQ-39, PDSS, BDI, FSS, UPDRS-I
				Control	69.6 ± 9.5 n=24				
Tunur et al., 2020 [73]		Pre-post -mixed methods	7	Google glass dance modules	69 ± 5.5 M: 3, F: 4	2–3	3+ modules/day, 3 weeks. Session length not reported	Not reported	MiniBEST, one leg stance, TUG, dual-task TUG, ABC, BDI, PDQL
Zafar et al., 2017 [82]		Pre-post design	88	Adapted Tango (PD)	68.4 ± 8 M: 13, F: 12	1–3	90 min, 2/week, 12 weeks	PD medication	Fear of falling, quality of life, composite physical function, MOCA, BDI, UPDRS-III, gait velocity, Impact on Participation and Autonomy Questionnaire
				Adapted Tango (non-PD)	82.3 ± 9 M: 14, F: 49				

Note: 6MWT, Six-Minute Walk Test; ABC, Activities-Specific Balance Confidence Scale; BBS, Berg Balance Scale; BDI, Beck Depression Inventory; EQ-5D, EuroQol-5 Dimension; FAB, Fullerton Advance Balance Scale; FOGQ, Freezing of Gait Questionnaire; FSS, Fatigue Severity Scale; HADS, Hospital Anxiety and Depression Scale; KFSS, Krupp Fatigue Severity Scale; MiniBEST, Mini-Balance Evaluation Systems Test; MBI, Modified Barthel Index; MOCA, Montreal Cognitive Assessment; PDQ-39, Parkinson's Disease Questionnaire-39; PDSS, Parkinson's Disease Sleep Scale; RCT, Randomised Controlled Trial; SDS, Self-Rating Depression Scale; SFHS-12, Short Form Health Survey-12; SS180, Standing-Start 180° Turn Test; Tinetti POMA Test, Tinetti Performance-Oriented Mobility Assessment Test; TUG, Timed Up and Go; UPDRS, Unified Parkinson's Disease Rating Scale.

**Table 3:** Method quality appraisal of included studies.

*Randomised controlled trials (PEDro Scale)*											
First author, year	Random allocation	Concealed allocation	Baseline-similar	Blinded participant	Blinded therapist	Blinded assessor	Measures for >85% sample	ITT	Between group analysis	Outcome measure data	Score/10
Duncan and Earhart, 2012 [62]	Y	Y	Y	N	N	Y	N	Y	Y	Y	7
Duncan and Earhart, 2014 [54]	Y	N	Y	N	N	Y	N	N	Y	Y	5
Hackney and Earhart, 2010 [88]	Y	N	Y	N	N	Y	N	Y	Y	Y	6
Hackney and Earhart, 2009 [61]	Y	N	Y	N	N	Y	N	N	Y	Y	5
Hackney et al., 2007 [59]	Y	N	Y	N	N	Y	N	N	Y	Y	5
Hashimoto et al., 2015 [67]	Y	Y	Y	N	N	Y	N	N	Y	Y	6
Kunkel et al., 2017 [55]	Y	N	Y	N	N	Y	Y	N	Y	Y	6
Lee et al., 2015 [57]	Y	N	Y	N	N	N	N	N	Y	Y	4
Lee et al., 2018 [63]	Y	Y	Y	N	N	Y	y	Y	Y	Y	8
McKee and Hackney, 2013 [66]	N	N	Y	N	N	Y	N	N	Y	Y	4
Michels et al., 2018 [30]	Y	N	Y	N	N	Y	Y	N	Y	Y	6
Poier et al., 2019 [64]	Y	Y	Y	N	N	Y	Y	Y	Y	Y	7
Rocha et al., 2018 [27]	Y	Y	Y	Y	Y	Y	Y	Y	Y	Y	10
Rios Romenets et al., 2015 [58]	Y	N	Y	N	N	N	Y	Y	Y	Y	6
Shanahan et al., 2017 [26]	Y	Y	Y	N	N	Y	N	N	Y	Y	6
Solla et al., 2019 [56]	Y	N	Y	N	N	Y	Y	N	Y	Y	6
Volpe et al., 2013 [65]	Y	Y	Y	N	N	Y	N	N	Y	Y	6

*Nonrandomised studies (JBI Appraisal Tool)*											
First author, year	Cause effect	Participants similar	Comparisons similar	Control group	Multiple measures	Follow-up	Consistent measurement	Reliable measurement	Statistical analysis		Score/9
Albani et al., 2019 [68]	Y	Y	N	N	Y	Y	Y	Y	Y		7
Batson, 2010 [77]	Y	Y	Y	N	N	Y	Y	Y	N		6
Blandy et al., 2015 [70]	N	Y	N	N	Y	N	Y	Y	Y		5
Dahmen-Zimmer and Jansen, 2017 [69]	Y	N	N	Y	Y	N	Y	Y	Y		6
Delextrat et al., 2016 [71]	Y	Y	N	N	Y	N	Y	Y	Y		6
Hackney and Earhart, 2009 [72]	Y	N	N	N	Y	N	N	Y	Y		4
Hackney and McKee, 2014 [28]	Y	N	N	N	Y	Y	N	Y	Y		5
Heiberger et al., 2011 [78]	Y	N	Y	N	Y	Y	Y	Y	N		6
Kalyani et al., 2019 [81]	Y	Y	N	Y	Y	Y	Y	Y	Y		8
Listewnik and Ossowski, 2018 [75]	Y	N	N	N	N	N	Y	N	Y		3
McGill et al., 2018 [85]	Y	Y	Y	Y	Y	N	Y	Y	Y		8
McKay et al., 2016 [80]	Y	Y	N	N	Y	Y	Y	Y	Y		7
McNeely et al., 2015 [74]	Y	Y	N	N	Y	N	Y	Y	Y		6
McRae et al., 2018 [87]	Y	N	N	N	Y	N	Y	Y	Y		5
Marchant et al., 2010 [79]	Y	N	Y	N	N	Y	Y	Y	Y		6
Rawson et al., 2019 [86]	Y	Y	Y	Y	Y	Y	Y	Y	Y		9
Seidler et al., 2017 [84]	Y	Y	N	N	Y	N	Y	Y	Y		6
Shanahan et al., 2015 [76]	Y	N	Y	N	Y	N	Y	Y	Y		6
Tillmann et al., 2020 [83]	Y	Y	Y	Y	N	N	Y	Y	Y		7
Tunur et al., 2020 [73]	N	Y	N	N	Y	Y	Y	Y	Y		6
Zafar et al., 2017 [82]	Y	N	Y	Y	Y	Y	Y	Y	Y		8

Note: Y=yes; N=no.

**Table 4:** Data analysis for randomised controlled trials.

Author (lead), year	Dependent variable	Outcome measure	Effect size	95% Confidence interval (CI)	Dose of intervention
Duncan and Earhart (2012) [62]: tango vs. usual care	Disability-motor	UPDRS-motor 3	−2.71	−3.40 to −2.02	1 hr class, 2/week, 12 months

Hackney and Earhart (2010) [60]: partnered tango vs. nonpartnered dance	Balance	Berg balance scale	−0.33	−0.96 to 0.30	1 hr, 2/week, 10 weeks
	Mobility	Timed up and go	0.52	−0.12 to 1.16	

Hackney and Earhart (2009) [61]: waltz/foxtrot vs. control argentine tango vs. control	Disability	UPDRS	WF: −2.61	−3.53 to −1.70	1 hr, 2/week, 13 weeks
			T: −2.44	−3.37 to −1.51	
	Balance	Berg balance scale	WF: 2.54	1.64 to 3.44	
			T: 2.52	1.57 to 3.46	
	Mobility	Timed up and go	WF: -1.74	−2.25 to −0.95	
			T: −2.14	−3.02 to −1.25	
	Endurance	6 minute walk test	WF: 1.86	1.05 to 2.66	
			T: 2.39	1.47 to 3.31	
	Freezing of gait	Freezing of gait	WF: 0.85	0.14 to 1.55	
			T: 0.76	0.03 to 1.49	
Tango vs. waltz/foxtrot	Disability	UPDRS	0.55	−0.17 to 1.27	
	Balance	BBS	−0.09	−0.80 to 0.61	
	Mobility	TUG	−0.75	−1.48 to −0.02	
	Endurance	6MWT	1.75	0.92 to 2.58	
	Freezing of gait	FOG	−0.08	−0.79 to 0.63	

Hackney et al. (2007) [59]: partnered argentine tango vs. group exercise class	Disability (motor)	UPDRS–Motor 3	1.53	0.51 to 2.55	1 hr, 2/week, 13 weeks
	Balance	Berg balance scale	3.52	2.09 to 4.96	
	Mobility	Timed up and go	−4.78	−6.54 to −3.01	
	Freezing of gait	Freezing of gait	1.56	0.54 to 2.59	
	Gait velocity	Gait velocity m/s	−1.01	−1.97 to −0.05	
	Dual tasking	Velocity of dual walking task m/s	−1.11	−2.08 to −0.05	

Hashimoto et al. (2015) [67]: PD dance vs. PD ex	Mobility	Time up and go	0.29	−0.40 to 0.99	Dance: 60 min class (dance), 1/week, 12 weeks Exercise: 60 min class (stretching, strengthening), 1/week, 12 weeks
	Balance	Berg balance scale	1.49	0.71 to 2.28	
	Disability	UPDRS	−0.89	−1.62 to −0.16	
	Depression	Self-rating depression scale	−0.18	−0.88 to 0.51	
PD dance vs. control (usual care)	Mobility	Time up and go	−0.22	−0.95 to 0.51	Dance: 60 min class, 1/week, 12 weeks
	Balance	Berg balance scale	1.05	0.27 to 1.83	
	Disability	UPDRS	−1.19	−1.98 to −0.40	
	Depression	Self-rating depression scale	−0.71	−1.46 to 0.04	

Kunkel et al. (2017) [55]: dance vs. control	Balance	Berg balance scale	−0.01	−0.62 to 0.59	1 hr, 2/week, 10 weeks
	Mobility	Timed up and go	0.37	−0.24 to 0.97	
	Endurance	6 minute walk test	−0.26	−0.87 to 0.34	
	Quality of life	PDQ-39	0.13	−0.47 to 0.73	

Lee et al. (2015) [57]: virtual reality vs. control	Balance	Berg balance scale	1.09	0.15 to 2.03	30 mins, 5/week, 6 weeks
	Activities of daily living	Modified Barthel index	1.12	0.18 to 2.07	
	Depression	Beck depression inventory	−1.30	−2.26 to −0.34	

Lee et al. (2018) [63]: Qigong dancing vs. wait list	Disability	UPDRS	−0.36	−1.00 to 0.27	60 min, 2/week, 8 weeks
	Quality of life	PD quality of life	0.55	−0.09 to 1.19	
	Balance	Berg balance sale	0.38	−0.25 to 1.01	
	Depression	Beck depression inventory	0.33	−1.22 to 0.06	

McKee and Hackney (2013) [66]: tango vs. education	Disability (motor)	UPDRS–Motor 3	−0.66	−1.45 to 0.12	90 mins, 20 sessions, 12 weeks
	Balance	Advanced balance scale	0.32	−0.45 to 1.09	
	Mobility	Timed up and go	−0.07	−0.83 to 0.70	
	Quality of life	PDQ-39	0.16	−0.61 to 0.93	
	Freezing of gait	Freezing of gait	−0.27	−1.00 to 0.53	

Michels et al. (2018) [30]: dance therapy vs. control	Disability (motor)	UPDRS–Motor 3	−1.32	-2.60 to −0.03	60 mins, 1/week, 10 weeks
	Disability	UPDRS–Total	−0.61	−1.81 to 0.59	
	Balance	Berg balance scale	1.32	0.03 to 2.60	
	Mobility	Timed up and go	−1.07	−2.32 to 0.18	
	Depression	Beck depression inventory	1.03	−0.21 to 2.27	

Poier et al. (2019) [64]: argentine tango vs. tai chi	Quality of life	PDQ-39	−0.14	−0.87 to 0.59	60 min class, 1/week, 10 weeks
	Satisfaction	BMLSS-life satisfaction	0.18	−0.55 to 0.91	

Rocha et al. (2018) [27]: argentine tango vs. mixed genre dance	Mobility	Timed up and go	−0.61	−1.49 to 0.27	In-person: 1 hour, 1/week Home: 40 mins, 1/week, 8 weeks
	Freezing of gait	Freezing of gait	0.26	−0.60 to 1.12	
	Balance	Berg balance scale	0.43	−0.44 to 1.30	
	Quality of life	PDQ-39	−0.75	−1.64 to 0.14	
	Disability (motor)	UPDRS 3 (R)	−0.01	−0.87 to 0.85	
	Disability (motor)	UPDRS 3 (L)	0.14	−0.72 to 0.99	

Rio Romenets et al. (2015) [58]: partnered tango vs. self-directed exercise	Disability	UPDRS-total	−0.50	−1.19 to 0.20	1 hr, 2/week, 12 weeks
	Disability (motor)	UPDRS 3	−0.60	−1.30 to 0.11	
	Quality of life	PDQ-39	0.11	−0.57 to 0.80	
	Mobility	Timed up and go	−1.00	−1.73 to −0.28	
	Mobility	Dual timed up and go	0.28	−0.41 to 0.97	
	Freezing of gait	Freezing of gait	−0.34	−1.03 to 0.35	

Shanahan et al. (2017) [26]: Irish set dancing vs. usual care	Disability (motor)	UPDRS 3	−1.13	−1.79 to −0.47	In-person: 1 hr, 1/week Home: 20 mins, 3/week, 10 weeks
	Endurance	6 minute walk test	0.13	−0.48 to 0.74	
	Quality of life	PDQ-39	0.00	−0.61 to 0.61	

Solla et al. (2019) [56]: Sardinian folk dance vs. usual care	Disability (motor)	UPDRS–Motor 3	−1.16	−2.11 to −0.21	90 mins, 2/week, 12 weeks
	Endurance	6 minute walk test	2.57	1.38 to 3.75	
	Balance	Berg balance scale	1.99	0.92 to 3.07	
	Mobility	Timed up and go	−1.81	−2.85 to −0.77	

Volpe et al. (2013) [65]: Irish set dancing vs. physiotherapy	Disability (motor)	UPDRS-motor	−0.99	−1.84 to −0.14	Dance: 90 min classes, 1/week, 6 months PT: 90 mins, 1/week, 6 months, 1 hr weekly home video
	Balance	Berg balance scale	0.81	−0.02 to 1.64	
	Freezing of gait	Freezing of gait	−1.45	−2.43 to −0.55	
	Quality of life	PDQ-39	−0.58	−1.43 to 0.23	

PDQ-39: Parkinson's Disease Questionnaire-39; PT: physiotherapy; UPDRS: Unified Parkinson's Disease Rating Scale. Analyses were for baseline and after intervention data within groups, unless otherwise specified.

**Table 5:** Data analysis for nonrandomised trials.

Author (lead), year	Dependent variable	Outcome measure	Mean difference (within group: baseline to after intervention)	95% confidence interval (CI)	Dose of intervention
Albani et al. (2019) [68] : home exercise + tango	Disability	UPDRS	−3.33	N/A	Home: 1 hr, 4/week, 5 weeks Group session: 2 hr, 1/week, 5 weeks
	Quality of life	PDQ-39	−3.57	N/A	

Batson (2010) [77] : modern dance	Mobility	Timed up and go	0.70	N/A	85 min class, 3/week, 3 weeks
	Balance	Fullerton Advanced Balance Scale	3.1	N/A	

Blandy et al. (2015) [70] : tango class	Quality of life	EurQol-5D	0.06 (median)	N/A	1 hr, 2/week, 4 weeks
	Depression	Beck Depression Inventory	4.50 (median)	N/A	

Dahmen-Zimmer and Jansen (2017) [69] : dance training versus karate	Depression	Hospital Anxiety Depression Scale	ES (between group) 0.61	−0.22 to 1.45	1 hr, 1/week, 30 weeks
	Wellbeing	SF-12	ES (between group) −1.02	−1.88 to −0.15	

Delextrant et al. (2016) [71]	Aerobic capacity	Mean heart rate	No significant effect of dance style on heart rate (*p* = 0.689).	N/A	Up to 1 hr, weekly, 6 sessions

Hackney and Earhart (2009) [72]: Argentine tango	Balance	Berg Balance Scale	2.80	N/A	1.5 hrs, 5/week, 2 weeks
	Disability (motor)	UPDRS-motor 3	−4.6	N/A	
	Mobility	Timed up and go	−2.0 (seconds)	N/A	
	Endurance	6-minute walk test	35.90 (metres)	N/A	

Hackney and McKee (2014) [28] : adapted tango	Balance	Berg Balance Scale	0.30	N/A	1.5 hrs, 2/week, for 12 weeks
	Mobility	Timed up and go	−0.19	N/A	

Heiberger et al. (2011) [78]: mixed dance for PD	Disability	UPDRS	8.2	N/A	1/week class, 1.5 hrs, 8 months
	Mobility	Timed up and go	0.7	N/A	

Kalyani et al. (2019) [81]: dance class versus control	Quality of life	PDQ39	ES (between group) 0.23	−0.46 to 0.91	1 hr, 2/week, 12 weeks
	Disability (ADL)	UPDRS-2	ES (between group) −0.13	−0.81 to 0.55	
	Depression	Hospital Anxiety Depression Scale	ES (between group) −0.71	−1.41 to 0.00	

Listewnik and Ossowski (2018) [75]: tango dance classes	Mobility	Tinetti Performance Oriented Mobility Assessment	2.15	N/A	70 mins, 2/week, for 12 weeks
	Mobility	Timed up and go	−1.72	N/A	
	Endurance	6-minute walk test	85.20	N/A	

McGill et al. (2018) [85]: ballet classes versus no dance	Gait	Step variability	ES (between group) 0.70	−0.03 to 1.42	1.25–1.5 hrs, 1/week, for 1 year
	Gait	Stride variability	ES (between group) 0.62	−0.10 to 1.34	
	Balance	Activities-Specific BalanceConfidence Scale	ES (between group) 0.24	−0.47 to 0.95	

McKay et al. (2016) [80]: adapted tango	Disability (motor)	UPDRS-motor 3	−2.90	N/A	90 mins, 15 lessons over 3 weeks
	Balance	Berg Balance Scale	3.80	N/A	
	Endurance	6-minute walk test	40.80	N/A	
	Mobility	Timed up and go	−1.10	N/A	
	Freezing of gait	Freezing of gait	0.10	N/A	

McNeely et al. (2015) [89]:tango vs. mixed dance	Disability (motor)	UPDRS-motor 3	ES (between group) −0.42	−1.41 to 0.57	1 hr group class, 2/week, 12 weeks
	Quality of life	PDQ-39	ES (between group) −0.25	−1.23 to 0.74	
	Mobility	Timed up and go	ES (between group) 0.2	−0.78 to 1.19	
	Mobility	Dual task timed up and go	ES (between group) 0.42	−0.57 to 1.41	
	Endurance	6-minute walk test	ES (between group) −0.39	−1.38 to 0.60	

McRae et al. (2018) [87]: dance classes	Quality of life	Short-Form Health Survey	3.84	N/A	1-2/week, 12 months (6 months–2yrs), session length unknown

Marchant et al. (2010) [79]: improvisation dance	Disability (motor)	UPDRS-motor 3	5.4	N/A	10 1.5 hour classes, 2 weeks
	Endurance	6-minute walk test	−3.8	N/A	
	Mobility	Timed up and go	−0.5	N/A	
	Balance	Berg Balance Scale	3.0	N/A	

Rawson et al. (2019) [86] tango vs. treadmill	Disability (motor)	UPDRS-motor 3	ES (between group) 1.35	0.83 to 1.87	1 hr class, 2/week, 12 weeks
	Endurance	6-minute walk test	ES (between group) 0.29	−0.19 to 0.76	
	Quality of life	PDQ-39	ES (between group) 0.44	−0.04 to 0.92	
Tango vs control (stretching)	Disability (motor)	UPDRS-motor 3	ES (between group) 1.07	0.56 to 1.57	
	Endurance	6-minute walk test	ES (between group) −0.80	−1.29 to −0.31	
	Quality of life	PDQ-39	ES (between group) 1.14	0.63 to 1.65	

Seidler et al. (2017) [84]: tele-rehabilitation group (tango versus in-person group)	Disability (motor)	UPDRS-motor 3	ES (between group) 0.22	−0.66 to 1.10	1 hr, 2/week, 12 weeks

Shanahan et al. (2017) [76] : Irish set dancing	Disability (motor)	UPDRS-motor 3	−2.0 (median)	N/A	In person: 1.5 hrs, 1/week Home: 20 mins, 2/week, 8 weeks
	Quality of life	PDQ-39	−4.03 (median)	N/A	
	Endurance	6-minute walk test	0.0	N/A	
	Balance	Berg Balance Scale	1.0	N/A	

Tillmann et al. (2020) [83] : Brazilian samba versus control		Beck Depression Inventory	ES (between group) 1.17	0.55 to 1.79	1 hr, 2/week, 12 weeks

Tunur et al. (2020) [73] : Google glass dancing	Mobility	Timed up and go	0.5	N/A	3+ modules/day, 3 weeks. Session length not reported
	Mobility	Dual task timed up and go	−0.5	N/A	

Zafar et al. (2017) [82] : adapted tango for PD versus AT for older adult	Participation	Participation and Autonomy Scale	ES (between group) 0.32	−0.20 to 0.83	90 min, 2/week, 12 weeks

Note: ES, effect size; PDQ-39, Parkinson's Disease Questionnaire-39; UPDRS: Unified Parkinson's Disease Rating Scale Analyses were for baseline and postintervention data within groups, unless otherwise specified.

## Data Availability

Data are available on request to the corresponding author.
